# Statistical Modeling of Transcription Factor Binding Affinities Predicts Regulatory Interactions

**DOI:** 10.1371/journal.pcbi.1000039

**Published:** 2008-03-21

**Authors:** Thomas Manke, Helge G. Roider, Martin Vingron

**Affiliations:** Max Planck Institute for Molecular Genetics, Berlin, Germany; Stanford University, United States of America

## Abstract

Recent experimental and theoretical efforts have highlighted the fact that binding of transcription factors to DNA can be more accurately described by continuous measures of their binding affinities, rather than a discrete description in terms of binding sites. While the binding affinities can be predicted from a physical model, it is often desirable to know the distribution of binding affinities for specific sequence backgrounds. In this paper, we present a statistical approach to derive the exact distribution for sequence models with fixed GC content. We demonstrate that the affinity distribution of almost all known transcription factors can be effectively parametrized by a class of generalized extreme value distributions. Moreover, this parameterization also describes the affinity distribution for sequence backgrounds with variable GC content, such as human promoter sequences. Our approach is applicable to arbitrary sequences and all transcription factors with known binding preferences that can be described in terms of a motif matrix. The statistical treatment also provides a proper framework to directly compare transcription factors with very different affinity distributions. This is illustrated by our analysis of human promoters with known binding sites, for many of which we could identify the known regulators as those with the highest affinity. The combination of physical model and statistical normalization provides a quantitative measure which ranks transcription factors for a given sequence, and which can be compared directly with large-scale binding data. Its successful application to human promoter sequences serves as an encouraging example of how the method can be applied to other sequences.

## Author Summary

The binding of proteins to DNA is a key molecular mechanism, which can regulate the expression of genes in response to different cellular and environmental conditions. The extensive research on gene regulation has generated binding models for many transcription factors, but the prediction of new binding sites is still challenging and difficult to improve in any systematic way. Recent experimental advances, notably high throughput binding assays, have shifted the theoretical focus from the prediction of new binding sites towards more quantitative models for the binding affinities of transcription factors, which can now be measured across whole genomes. Therefore we have developed a biophysical model which accounts for much of the observed variation in binding strength. Here we extend this framework to model not just the binding affinity, but also its distribution in various sequence backgrounds. This enables us to compare predicted affinities from different transcription factors, and to rank them according to their normalized affinity. What are the biological implications of such a ranking? We have demonstrated that many known associations between transcription factors and their respective targets appear as strong interactions. This provides a rationale to predict, for any given promoter region, those transcription factors which are most likely to be involved in its regulation.

## Introduction

Several experimental advances in the study of gene regulation have highlighted the fact that transcription factors have a certain affinity to all DNA regions, as evidenced by many experimental techniques, such as DNAse footprinting [1], gel-shift assays [2], SELEX [3], and more recently genome-wide chromatin-immunoprecipiation (ChIP-chip) [4]. Traditionally, such binding data has been subjected to a threshold, in order to distinguish binding sites from non-binding sites. Sequence-based binding models often rely on a collection of such binding sites and aim to generalize the available information in terms of a “regulatory code.” The most common approach seeks a statistical description of the binding preferences in terms of a motif matrix, which records the observed nucleotide frequencies at each position in an alignment of known binding sites. The classical work of Berg and von Hippel [5] provides a simple biophysical model to convert the motif matrix into a matrix of mismatch energies, which contribute independently to the overall binding energy of a protein-DNA complex. With this model, it is possible to assign binding energies to any sequence site of width *W*. While it is common practise to classify also novel sites as binding or non-binding, there is nothing fundamental about such a distinction, which depends on a threshold of the binding energy or a related score.

We have recently shown that such a threshold is not necessary to understand and to quantitatively model a large amount of binding data from ChIP-chip experiments in yeast [6]. In fact, the imposition of a threshold on both the experimental data and in-silico predictions hinders the direct comparison of the theoretical model with experimental observations. Therefore we have introduced a new method, called TRAP for TRanscription factor Affinity Prediction, which has shifted the focus from the prediction of right or wrong binding sites to quantifying the differences between weak and strong binding. In our earlier work [6], we provided a parametrization of the TRAP-model which can be applied to any motif matrix, even in the absence of large-scale data, such as ChIP-chip screens.

While the TRAP-model allows to rank sequence regions according to their different affinities for a given transcription factor, it cannot always be applied to compare different transcription factors for a given sequence. This is because different transcription factors can have very different specificities, i.e. different distributions of affinities. In this paper we aim to remedy this situation by providing a proper normalisation, such that the binding affinity of different factors can be directly compared with each other. To this end we define a statistical score (p-value), which assigns the probability of observing a certain affinity or higher in a given sequence background. Here the goal is not to set some significance threshold, but rather to normalise an observed affinity in the light of a random sequence model, and to give a statistical meaning to the statement that one factor binds stronger than another.

In Section (2), we briefly review the TRAP model and introduce our notation. In Section (3) we derive the exact affinity distribution for an arbitrary motif matrix and for sequence backgrounds where all nucleotides are drawn independently from the same distribution (iid). We then show that this distribution can, to a large extent, be parametrized by an extreme value distribution, and that this effective characterisation can also account for the affinity distribution in non-iid sequences with variable GC-content. We also compiled a complete parametrization for 762 TRANSFAC matrices which can be used for promoter regions of variable length, and without having to repeat the statistical modeling. To highlight the biological relevance of our approach, we present a realistic application of our method to human promoter regions. We show that many known regulatory interactions can be infered based on the high affinity of the associated transcription factor to the relevant promoter region.

## Materials and Methods

### Binding Site Descriptions

For many transcription factors, motif matrices have been constructed from alignments of known binding sites. Here we rely on the curated results and matrix descriptions provided by the TRANSFAC database [7]. A motif matrix *M* = (*M_w,_*_α_) records how frequently a nucleotide α has been observed at position *w* within the alignment. We take all 762 matrices which were derived from small-scale experiments and dedicated *in vitro* studies in several different species. In order to set a maximal mismatch energy, we added a pseudo-count of π = 1 to each element in the count matrices.

### The TRAP model

**Figure 1:**
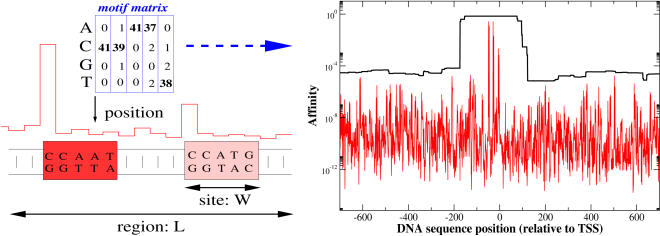
TRAP approach. The left-hand side illustrates how a given motif matrix (*W* = 5) is scanned against a longer DNA sequence region of length L. At each position the binding energy for the adjacent site is calculated as in Eq. (3), which assumes independence of all positions within a site. The binding energy is converted into a local affinity using Eq. (1) and the parametrization from [6]. This results in the schematized red curve of position-dependent binding affinities. Two selected sites are shown as red boxes, because they correspond to relatively high affinities in this toy example. In our framework we do not annotate them as “hits,” but rather sum the different contributions from all possible positions (and strand orientations) – see Eq. (4). This gives rise to a total affinity of the sequence region with length L. Initially this approach was developed to rationalize the ChIP-chip data, where L corresponds to the experimental fragment length [6]. On the other hand, the summation in Eq. (4) also amounts to a smoothing of the noisy binding signal over larger sequence regions. This is shown on the right-hand side, where the affinity of transcription factor SRF (*W* = 15) is calculated around its own promoter region. Here the red line denotes the local affinities which fluctuate strongly, and the black curve denotes the combined affinities for longer regions of length *L* = 500.

In our earlier work [6] we were primarily interested in comparing different promoter sequences with respect to their binding affinities for a fixed transcription factor. This has been successfully applied to account for much of the observed variation of binding strength in ChIP-chip experiments. Here we will briefly review the TRAP model and its biophysical background. The key concepts are also illustrated in Figure 1. First consider many copies of some DNA site, *S_l_*, which extends from sequence position *l* to *l*+*W*−1. In the following we assume that the fraction of such sites, which are bound to a given transcription factor, *T*, can be calculated using an equilibrium approach. We call this fraction the local affinity, *a_l_*,
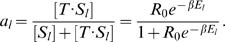
(1)


Here *R*_0_ is a positive, sequence-independent parameter, and *E_l_* is a site-dependent *mismatch energy*. The latter is chosen such that *E_l_* = 0 for the site with the highest possible affinity (consensus site) and *E_l_*>0 for sites that deviate from the consensus. Following the model of Berg and von Hippel [5], the mismatch energy for many transcription factors can be calculated as follows. Let *M* = (*M_w,_*_α_) be a *W*×4 motif matrix from an alignment of known binding sites, where *w* = (1…*W*) are the positions within the alignment, and α denotes one of the four nucleotides, α = (*A*, *C*, *G*, *T*). The matrix entries are the nucleotide counts in this alignment. Now one can match this motif matrix against any other site of width *W* and define the position-specific contributions to the mismatch energy as
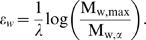
(2)


Here α refers to the actual nucleotide of the sequence at position *w* within the current site, *M_w,max_* is the maximal entry in column *w* of the matrix, and λ is a scale-parameter. The key assumption of the model by Berg and von Hippel is that all ε*_w_* contribute independently to the overall mismatch energy at site *S_l_* [8]

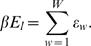
(3)


From the above ingredients we determine the expected number of transcription factors bound to a longer DNA sequence region of length *L*. We call this number the total affinity, *A*, which can be obtained by summing up all contributions from both strand, *a_l_*, and anti-strand sequences, 
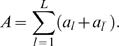
(4)


For a more detailed exposition we refer the reader to our earlier work [6], where we have also included a correction term for palindromic motifs. The reader should be aware that the TRAP-parameters, λ and *R*_0_, were determined from large-scale ChIP-chip data with many different factors and cellular conditions. Importantly, our earlier work also revealed, that they could be determined simultaneously for all transcription factors, and need not be tuned individually. The general TRAP model is then defined by λ = 0.7, and ln(*R*_0_) = 0.585*W−5.66. Notice that ln(*R*_0_) also depends logarithmically on the transcription factor concentration, but this dependence is much weaker than the linear dependence on the motif width, *W*. We want to emphazise that our simple model does not provide the most accurate description for each individual factor and every cellular condition. If large-scale binding data is available, the TRAP model could be improved by further tuning the parameters for each specific setting, but here we aim for generality.

### Derivation of the Exact Distribution of Affinities from a Motif Matrix

Given the general TRAP-model with fixed parameters, the affinity of a transcription factor to a specified sequence region depends only depends only on the sequence composition and the matrix description of the factor. For simple sequence models, it should therefore be possible to calculate the affinity distribution for any matrix exactely. For simplicity we assume a sequence background with a given GC-content, i.e. a given single-nucleotide distribution (π*_A_*, π*_C_*, π*_G_*, π*_T_*). According to Eq. (2), the distribution of nucleotides induces a discrete distribution of mismatch energies, 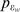. Next we determine the probability distribution of the overall energy, which is determined by independent contributions of ε*_w_* (Eq.(3)). Therefore the probability probability distribution *p_E_* can be calculated by convolution of 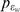 over all positions *w* within the motif of width *W*. It is a standard procedure and technically simple to calculate this convolution using Fourier transformation
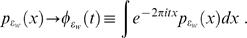
(5)


It follows from the convolution theorem that the Fourier transformation of *p_E_* can be written as a simple product of Fourier transformations [9]

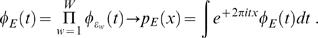
(6)


Here the final step denotes the inverse Fourier transformation to revert back to the orginal representation This derivation is completely analogous to the approach by Staden [10], who calculated the score distribution using moment-generating functions. Our biophysical model has the added advantage that all energies, *E*, can be directly converted into local affinities (Eq.(1)). Accordingly, the probability of observing a certain energy *E* is identical to the probability of observing the corresponding affinity *a* = *a*(*E*). We denote this simple map by *p_E_*→*p_a_*.

As before we consider the Fourier representation of *p_a_*

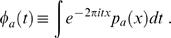
(7)


Now it is straightforward to derive also the distribution of total affinities for a sequence region with length *L*, as defined in Eq. (4). Here we assume that the local affinities of neighbouring sites are independent from each other and identically distributed. This reduces the analysis to a repetition of the above approach in terms of Fourier transformations
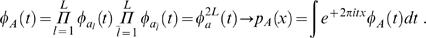
(8)


In practice we utilize a Fast Fourier Transformation (FFT), to evaluate all the above integrals numerically.

## Results

### The Exact Distribution of Affinities from a Motif Matrix

**Figure 2:**
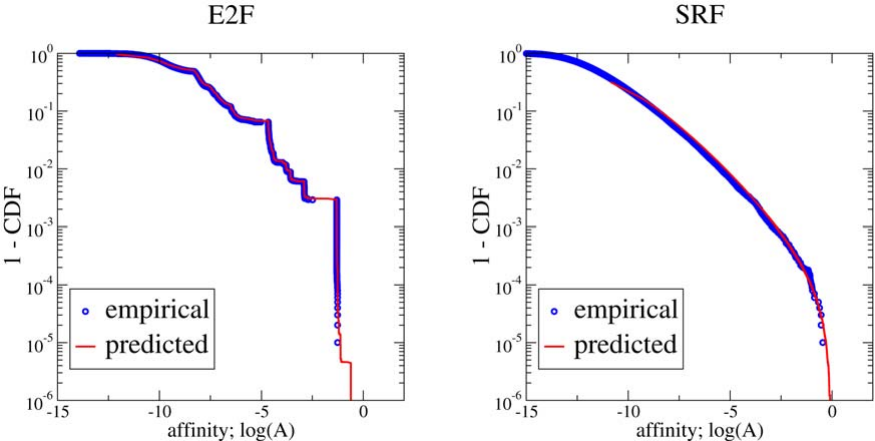
Empirical affinity distribution and theoretical prediction. Here we compare the empirical distribution (blue circles) of affinities calculated on 100,000 random sequences of length *L* = 100 and the predicted cumulative distribution (red line) derived from Eq. (8). Due to the wide range of affinities and corresponding probabilities, we present the distributions on a double-logarithmic scale. The left plot is for transcription factor E2F. It illustrates that, in general, we cannot expect to find a simple parametrization of the affinity distribution, although it can be understood from first principles. On the other hand, the affinity distribution for SRF (right plot) is better behaved and can be well described by a generalized extreme value distribution.

For a simple sequence model of identical and independently distributed nucleotides (iid), it is possible to calculate the exact distribution of affinities as described in the Methods section. In Figure 2, we show the cumulative distribution, 
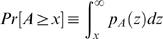
, for two particular matrices and a region of *L* = 100 basepairs. For ease of presentation, we choose a double-logarithmic scale for this plot. Also in the remainder of the paper we will always work with logarithmic affinities, log (*A*).

Notice that our theoretical results completely agree with the empirical distribution of calculated affinities for a random background sequence with fixed GC-content. The figure also illustrates that, in general, the distribution is not easily parametrized and certainly not normally distributed. A similar point has been made previously in the context of score distributions [11]. The step-like behaviour of many cumulative distributions is a reflection of the discrete nature of binding energies which are derived from discrete count matrices.

The reader should be reminded that for the above derivation we have assumed that all contributions to the total affinity are independent of each other. This is consistent with the assumptions of the physical model, Eq. (3) and Eq. (4). There are several matrices, for which this assumption does not hold as they possess a high degree of self-similarity, e.g. *CF*2*II* with consensus motif GTATATATA. Nevertheless we find only small deviations of the predicted distribution from the empirical data, which is negligible for practical purposes. One may try to define more complicated statistical models to account for such self-similarities, but fundamentally, this issue should already be addressed at the level of Eq.(4), which requires correction terms to account for preclusion and competition of factors for binding sites. This question has been addressed in our earlier work [6]. In the following, we do not pursue any more complicated models, but rather aim to find a simpler and effective description of the distribution function with only a few parameters.

### Parametrization for iid Background Sequences

While it is satisfying to obtain a theoretical expression for the exact distribution of affinities, this is not particularly convenient for practical purposes as the full distribution function would have to be stored for different region sizes, L, and different GC-contents. Moreover, ultimately we will seek to model promoter sequences which are not iid, but tend to have highly variable GC-content. Therefore we are now searching for a convenient parametrization, which can be used efficiently in practice. We recall the explicit goal of this project, which is to provide a proper normalization of binding affinities, such that different binding factors can directly be compared with each other. From the previous section is is apparent that a simple parametrization will not be possible in general. The best one can hope to achieve is an effective parametrization, which is indistinguishable from the empirical distribution, at some level of accuracy. In this section we will compare the ability of several standard parametrizations to model the distribution of 1000 affinities for several different sequence backgrounds.

To quantify the overall “failure rate” of a parametrization, we determined the number of matrices for which the Kolmogorov-Smirnov test discriminates between the parametrization and the empirical distribution at the level of *p*_KS_<0.05. Notice that this choice of threshold is arbitrary and that we do not consider *p*_KS_ to be proper p-values, as the parametrization was obtained from a best fit to the data. However, we applied the same procedure consistently to all setups, which still allows us to compare the relative performance of different parametric models for different sequence backgrounds.

First, we generated 1000 random sequences (*L* = 2000) and systematically tested the empirical affinity distributions of all 762 TRANSFAC matrices against the log-normal distribution. However in 553/762 cases the fitted distribution model was significantly different from the empirical distribution, as assessed by the Kolmogorov-Smirnov test (*p*_KS_<0.05). This indicates that for most matrices the log-normal parametrization *P*(*x* = log(*A*)|μ, σ) is not appropriate.

Next we tested the same random data against a generalized extreme value (GEV) distribution with 3 parameters (*a* = shape, *b* = scale, *c* = location) [12]

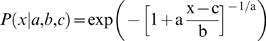
(9)


This parametrization is motivated by the fact that the total affinity *A* is often dominated by the highest affinities in the region under consideration, or only a few terms in Eq. (4).

**Figure 3:**
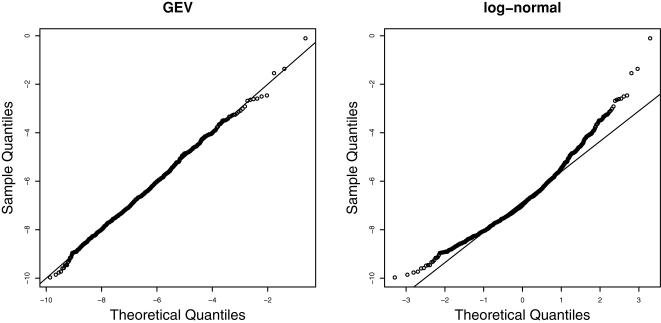
The GEV distribution provides a better fit to empirical data than a log-normal distribution. The left figure shows the QQ-plot of the numerical data against the fit to a generalized extreme value distribution, Eq. 9. This example is for one matrix (GAL4_01), for which we calculated affinities for 1000 random background sequences of length *L* = 1000. The GC-content was fixed at 50%. In this case we obtain *a* = −0.07(2), *b* = 1.15(3), *c* = −7.36(4) which is an appropriate parametrization given the data, *p*_KS_ = 0.85. It should be contrasted to the best log-normal fit (right), which gives (μ = −6.77(4), σ = 1.35(3)), but this parametrized distribution is significantly different from the empirical data, *p*_KS_ = 0.0003.

Indeed, the GEV distribution accounts for the bulk of the empirical data very accurately. Only for 61/762 matrices this parametrization is not compatible with the actual distribution of 1000 simulated affinities (*p*_KS_<0.05). Such failures can be exemplified by the case of E2F, for which we did not find any suitable parametrization, left plot of Figure 2. However, for the majority of all matrices the GEV distribution presents a very appropriate model, as illustrated by the example for Gal4 in Figure 3 and summarized in Table 1.

**Table 1:** Failure rates of different parametrizations and several sequence background models.

Model	Logn(μ, σ)	Gumbel(*b*,*c*)	GEV(*a*,*b*,*c*)	GPD(*a*,*b*,*u*)
Rand(GC = 0.5,L = 100)	644	197	49	41
Rand(GC = 0.5,L = 1000)	614	213	60	36
Rand(GC = 0.5,L = 2000)	553	272	61	30
Rand(GC = 0.5,L = 10000)	364	447	44	14
Rand(GC = 0.4,L = 2000)	558	275	60	30
Rand(GC = 0.2–0.8,L = 2000)	608	697	460	30
Genomic ( 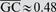 ,L = 2000)	445	553	157	27
Promoters ( 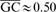 , L = 2000)	467	608	178	24

**Figure 4:**
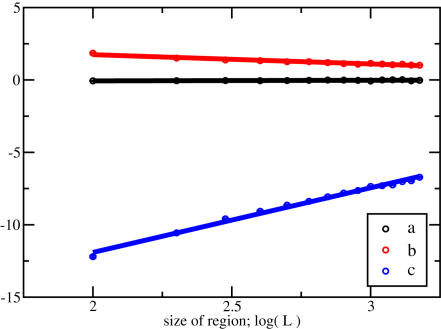
Regression of GEV parameters. Based on our analysis of Eq. (12), we were led to a regression analysis for the parameters of the generalized extreme value distribution against the logarithm of the region size, log *L*. As in Figure 3 we use the example of GAL4, but the analysis was done for the complete set of 762 TRANSFAC matrices. The regression for the three parameters gives: (*a*_0_, *a*_1_) = (−0.14, 0.04), black line with *p_F_* = 0.04, (*b*_0_, *b*_1_) = (3.03, −0.64), red line with *p_F_* = 2×10^−10^, (*c*_0_, *c*_1_) = (−20.8, 4.46), blue line with *p_F_* = 4×10^−15^. As was mentioned in the main text, the shape *a* of the GEV is almost independent of *L* and small and negative. This holds true for all TRANSFAC matrices.

Here we summarize the performance of the parametrizations in terms of the number of TRANSFAC matrices (out of 762) for which the Kolmogorov-Smirnov test can discriminate between the empirical distribution and its parametrization. This Table illustrates that the GEV distribution with 3 parameters has far greater explanatory power (a lower “failure rate”) than simpler distributions with only two parameters (log-normal, Gumbel). If only the upper tails of the distribution are to be modeled, then the location parameter, *c*, can be traded for a threshold, *u*, above which a generalized Pareto distribution (GPD) applies. These observations are largely independent of the length, *L*, of the region for which the affinities are calculated, although the parameter values do change, as shown in Figure 4. Although the power of the GEV parametrization deteriorates for non-iid backgrounds, it is still much better than the other parametrizations. This is shown in the last three rows of the table for random sequences with simulated GC-variability ∈[0.2, 0.8], genomic DNA, and human promoter regions.

While the addition of one extra parameter seems like a small prize to pay for the much better coverage, one should remember that all parameters depend on the length, *L*, of the region. Now we will show that the *L*-dependence can also be parametrized. Let (*a*(*L*),*b*(*L*),*c*(*L*)) denote a given parameter configuration. For the mean of the distribution we can write
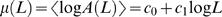
(10)

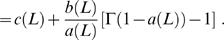
(11)


Here the first line follows from 〈*A*/*L*〉 = constant, while the second line is a specific property of the GEV distribution, where Γ denotes the Gamma function [12]. Furthermore, we observe that *a*≪1 for all our fits, or Γ(1−*a*)≈1. This motivates the following regression for the location parameter *c*(*L*)
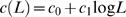
(12)


The coefficients (*c*_0_,*c*_1_) need to be determined for each matrix separately. To this end we extended our numerical simulations to a range of different region sizes, *L* = 100–1000, and determined the best parameters by linear regression against log *L*. This is shown for one matrix, GAL4_01, in Figure 4, where we also present the analogue behaviour for the other parameters (scale b(*L*) and shape *a*(*L*)). We find a good regression also for all other matrices. Notice that *a*(*L*) is almost flat (*a*_1_≈0) and close to zero for most matrices. In the light of this observation we also considered fits to a Gumbel distribution with one less parameter (*a*→0). This resulted in many (>200) unacceptable parametrizations, see Table 1. Therefore we disregarded this possibility.

To summarize this section, we succeeded in deriving an efficient parametrization of the affinity distribution for sequences with fixed GC-content and for different region sizes, *L*.

### Parametrization for Non-iid and Genomic Sequences

The above results indicate that for a simple background model one can predict the exact distribution of affinities and, to a large extent, find a relatively simple parametrization in terms of a GEV distribution (Eq. 9). The more relevant question for possible biological applications is, of course, whether the same is possible for genomic DNA with variable GC-content. To answer this question we repeated the above analysis for three non-iid background models:

- randomly generated sequences with variable GC-content,
- genomic sequences (from human chromosome 1).
- human promoter sequences of 2000 bp centered around the transcription start site

The last choice was motivated by the specific application discussed in a subsequent section. From now on, we will always consider regions of fixed size *L* = 2000 basepairs, unless stated otherwise. While the coverage of the GEV-model does not depend on the size of the region, a variable GC-content significantly increases the number of matrices for which the parametrization cannot be considered appropriate. This is shown in Table 1, where we give the failure rate for different parametrizations and backgrounds. For the GEV model, the discrepancy is most apparent in a rather extreme background of 1000 random sequences, where the variable GC-content was chosen from a uniform distribution over [0.2,0.8]. Unsurprisingly, the GEV distribution does not properly account for such extreme variability, as many matrices (and their affinities) are very sensitive to changes in GC-content.

For the second background we considered a 2 Mbp region from human chromosome 1, which also has variable GC-content with an average of 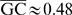. The fluctuations in GC are however much less severe than in the previous model and the GEV-model is again appropriate for the majority of matrices.

Finally, we consider a background of 1000 human promoters (2000 bp centered around randomly chosen transcription start sites). Their average 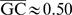, is close to our iid background model, but with large deviations from the average due to specific promoter properties and CpG islands. While the GEV distribution cannot reliably account for the promoter-specific distribution of 178 TRANSFAC matrices, the remainder of 584 matrices can be modeled quite accurately by GEV, at the given level of significance (*p*_KS_ = 0.05). This is much better than any other class of distributions we have tested. We provide a complete list of the parameters for 762 TRANSFAC matrices as supplementary material (Text S1) [13].

### Parametrization of the Tails of the Distribution

So far we focused on the derivation and parametrization of the whole distribution for small and large affinities. However, in practice we are hardly ever interested in affinities which are small in the context of some background model. Therefore the more crucial question is whether the tails can be modeled appropriately. To this end we invoke a theorem from Extreme Value Theory which states that under very general assumptions the tails of the distribution, above some threshold *u*, follow a generalized Pareto distribution (GPD)
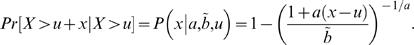
(13)


Of course, the threshold *u* is somewhat arbitrary, but should be chosen large enough for the limit theorem to be applicable. In this regime the GPD reduces to two parameters, which can be determined by fitting the tails of the empirical distribution. Here we defined the threshold *u*, such that the upper tails contain 5% of the distribution. As can be seen from Table 1, the GPD-model accounts for the distribution of a large fraction of all matrices. While we have manually inspected the threshold dependence for selected matrices, we did not optimize the threshold for each matrix separately. Such a procedure, may further improve the observed coverage, but it would be very cumbersome to do for 762 matrices.

### Application

Now we show how to apply our results in a realistic setting. Consider, as an example, the promoter region of SRF, which we take to be a 2000 bp region centered at the transcription start site. The biologically relevant question is to decide which transcription factors are most likely to regulate the activity of SRF. We want to stress that our approach, as well as any other sequence-based approach, cannot answer this question in any fundamental way, as we only characterize the binding strength of a factor, but not its regulatory potential. For this more refined question one ultimately needs to invoke additional information, such as expression data [14],[15] or evolutionary conservation [16],[17].

In the absence of such functional data, information on the binding strength may still guide biological investigations – an approach which is also taken for the analysis and interpretation of ChIP-chip data. However, a simple ranking of all known transcription factors according to their predicted binding affinity would not be very meaningful either. In our example, those factors with the highest calculated affinity are DFD, MINI20 and others, all of which have very high base affinities, but which are not specific to the promoter region of SRF.

In order to discriminate those factors which have high affinity specifically for the SRF promoter but not the background, we invoke the statistical approach and the background model defined above by the set of human promoters. In Table 2 we list the top scoring matrices along with their normalized scores. As can be seen, many of them relate to the same transcription factor SRF, which is well-known for its autoregulatory capacity. Clearly the motif descriptions are not independent, but here we do not aim to resolve such redundancy and leave it as a postprocessing step of our analysis.

**Table 2:** Ranked TRANSFAC matrices based on their affinity to the SRF promoter (*L* = 2000).

Not Normalized		Normalized	
Matrix-ID	Affinity	Matrix-ID	−log[*p*_GEV_(Affinity)]
I$DFD_01	51.53	V$SRF_*Q*5_02	3.02
V$MINI20_*B*	10.07	V$SRF_*C*	2.89
V$MINI19_*B*	9.40	V$SRF_*Q*4	2.77
I$ADF1_*Q*6	8.18	V$SRF_*Q*5_01	2.72
V$AP2ALPHA_01	4.85	F$CAT8_*Q*6	2.48
V$ETF_*Q*6	3.94	V$SRF_*Q*6	2.18
V$MUSCLE_INI_*B*	3.60	F$REB1_*B*	1.95
F$FACBALL_*Q*2	3.53	V$OCT_*C*	1.89

On the left site we give the top matrices, which are naively ranked according to their calculated affinity. On the right site we show the same number of top-ranking matrices, ranked according to the corresponding p-value from the GEV-parametrization.

As a further example, we also considered the promoter region of another transcription factor, E2F, which is a known auto-regulator [18]. We use an identical setup as described above and determined those transcription factors with the highest affinity to the E2F-promoter after normalization. Also for this gene we find a strong binding of the transcription factor E2F to its own promoter (Table 3). As a final example, we turn to a gene which has a strong tissue-specific expression domain. The serine protease inhibitor A1 (α1-antitrypsin) is part of the larger serpin cluster of genes which is almost exclusively expressed in liver, for both human and mouse. Our analysis reveals a particularly strong binding of the liver-specific factor HNF1, which has indeed been known to be involved in the regulation of α1-antitrypsin [19].

**Table 3:** E2F-promoter and A1A-promoter.

E2F		α1-antitrypsin	
Matrix-ID	−log[*p*_GEV_(Affinity)]	Matrix-ID	−log[*p*_GEV_(Affinity)]
V$E2F_03	2.87	V$HNF1_01	2.97
F$REPCAR1_01	2.70	V$HP1SITEFACTOR_*Q*6	2.77
V$E2F1_*Q*3_01	2.36	V$AP1_*Q*4	2.31
V$E2F_*Q*3_01	2.35	V$HNF1_*Q*6	2.24
V$E2F_*Q*4_01	2.31	V$AP1_*Q*2	2.24
V$E2F_01	2.29	V$AP1_*Q*6	2.18

As in Table 2 we list those matrices with the strongest normalized binding affinity to two well-known promoters. E2F is a known auto-regulator [18], while α1-antitrypsin is a liver-specific gene, which is known to be regulated by HNF1.

While the regulatory mechanisms for all those genes are likely to involve additional sequence elements and transcription factors, it is encouraging that some of the known key players can be detected by our method. We should also point out that we have assumed a scenario of maximal ignorance. Frequently one may already have a list of transcription factors among which to choose the one with the highest relative affinity. For example, in the case studies above, one may have excluded all non-vertebrate factors, or further restricted them to those which share an expression domain with the gene in question, if this information is known. Here this was not even necessary and it bodes well for the applicability of our framework to other promoters.

**Figure 5:**
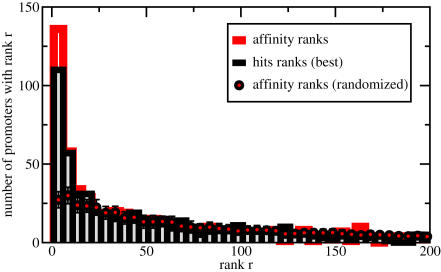
High affinities predict known regulatory interactions. For each of 567 human promoters we determine the ranks of the transcription factors which are known to bind this promoter, based on our affinity ranking. The red histogram shows the distribution of these ranks. For example, 136 of 567 promoters have a known regulator assigned within the top 5 of 554 vertebrate Transfac matrices. For 50 promoters a known regulator is also the top candidate within our ranking scheme. This should be compared to the red circles, which have been obtained from the same analysis, but with reshuffled promoter regions. The reshuffling was done 100 times to determine the standard deviation, which is shown as error bars. Finally we also ranked the prediction from a traditional approach which assigns a number of binding sites to each promoter region. Notice that many transcription factors can have an identical number of binding sites, which leads to ambiguous ranking schemes. To be conservative, we always assign the best possible rank (histogram shaded in grey). Compared to the traditional approach our method identifies a larger number of known regulatory interactions.

To assess this point more quantitatively, we retrieved from ENSEMBL (version 45 [20]) the promoter sequences (2000 bp around the TSS) of 567 human genes, which have known binding sites according to the TRANSFAC database v11.1 [7]. We also required that at least one of the associated transcription factors has a matrix description which enables a treatment by our affinity analysis and normalization, as described above. For each of the 567 promoters, we compare and rank all 554 vertebrate motif matrices, and determine the rank of the matrix for the known binding factor. In cases where known factors are represented by multiple matrices, we take the one with the best rank. In Figure 5 we show that there is indeed a large fraction of promoters, for which the associated transcription factors rank highly according to their normalized affinity. Specifically, we find that for 50 out of 567 promoters the top-ranking matrix corresponds to a known regulator, as exemplified by the earlier examples. To the extent that experimentally known binding sites are functional, our analysis also illustrates that in many cases binding strength is suggestive of functionality.

Next we want to assess the significance of these findings. One might expect a shift towards higher ranks, simply because we always use the best matching factor, if more than one is known to regulate a certain gene. To account for this effect we reshuffled the associations between factors and promoters, while retaining the precise distribution of factors per promoter. Then we repeated our analysis on 100 such randomized sets and obtain 100 corresponding histograms. From this we determine the average histogram and the standard deviation, which are shown as red circles with error bars. While there is indeed a slight increase towards higher ranks, it is also clear from Figure 5 that the observed increase is highly significant.

Now we want to compare these results with what one would obtain from a traditional hit-based analysis. To this end we employ the annotation method which was introduced by Rahmann *et al* [11]. In this approach one defines a score threshold such as to balance the rate of false positive and false negative hits. In doing so we assign to each of the 567 promoters and every vertebrate TRANSFAC matrix the number of significant hits, which are observed above the “balanced” threshold. Notice that many factors can have the same number of hits, and some may have none. This makes the hit-based ranking somewhat ambiguous – in contrast to our affinity-based ranking, which assigns a positive and real number to each combination of promoter and transcription factor. Here we take a favourable approach to the hit-based predictions, and assign to a known regulator always the best possible rank. Consider, as an example, the 2 kb promoter region around the transcription start of the Nod1 gene. This gene encodes a protein which can sense invasive bacteria and initiate an inflammatory response [21]. It is also known to be regulated by IRF1 [22]. In fact, the corresponding motif ranks highest according to the normalized affinity, and the matrix has 5 hits in the promoter region. However, there are many other factors which have also five or more significant hits. According to the hit-based method the IRF1 transcription factor would rank between 68 and 87. For our conservative comparison we would assign the higher rank: 68. The results from a systematic analysis over all promoters with known regulators are shown in Figure 5 (histogram shaded in grey). While the hit-based method also recovers many known regulatory links, it is apparent that our quantitative approach predicts more biologically meaningful associations.

In our earlier study [6] we had already observed that the affinity-based ranking of sequences for a given factor is only little affected by changes in the global TRAP parameters (λ, *R*_0_). In the current context, we have repeated such an analysis also for the ranking of transcription factors. We find that the results from Figure 5 are almost identical if λ is changed from λ = 0.7 to 1.0, or if ln(*R*_0_) is artificially increased by 20% (Figure S1). This indicates that our ranking scheme provides a robust approach to quantify the binding strength and to discriminate transcription factor from each other.

## Discussion

In this paper we adopted a novel approach to the modelling of protein-DNA interactions. Rather than identifying transcription factor binding sites, we quantify the affinity of a transcription factor to any given sequence region. In contrast to the traditional approach, we do not seek a specific threshold, and we do not study “hits” of transcription factors. Instead, we are seeking an appropriate normalisation, which allows us to compare the affinities of different transcription factors directly with each other. This is similar in spirit to the different normalisation procedures which are currently applied to experimental ChIP-chip data [23],[24], for which the TRAP-model was originally designed [6].

In our earlier work we were mostly concerned with the ranking of different sequences for a given factor and we derived an optimal model to achieve just this. Here we addressed the more challenging task to compare the affinities of different factors for a given sequence. This requires an understanding of the affinity distribution, which can be used to define a comparable score (a p-value). We have shown that, for a simple background model, the exact distribution of affinities can be predicted directly for any matrix. For a given sequence, all transcription factors with matrix descriptions can be ranked according to how strongly their affinity deviates from its expected value.

While the exact analysis can in principle be repeated for uniform sequences of all lengths, we also provide a relatively simple parametrization (GEV-model), which is applicable for more than 90% of all matrices, and in which the length dependence can also be accounted for, through a regression analysis. Moreover, the GEV parametrization can also account for the distribution of affinities from sequences with variable GC-content, as long as the variability is not too strong. To demonstrate our approach in a realistic stetting, we have applied this normalization to human promoters with known binding sites. We find that matrices of known transcription factors tend to rank highly according to their normalized affinity. This has been illustrated by the example of the SRF promoter, which yielded a clear suggestion for a known auto-regulatory loop, i.e. a strong relative binding of SRF to its own promoter. Remarkably this link could be established without invoking any prior knowledge on the set of relevant transcription factors, and without sequence conservation.

We want to stress that even the best parametrizations used in our work leave room for improvement. While we have made an extensive effort to derive a simple characterization which is appropriate for most matrices, we clearly traded accuracy for efficiency (small number of parameters). More specifically, the GEV-model should not be used to estimate p-values very accurately. Instead, it represents an effective distribution which is appropriate at a certain level of granularity. As the distribution models in our study were derived from the empirical distribution of 1000 measured affinities, we do not expect accuracies better than ∝10^−3^, even for those matrices for which we consider the GEV-model appropriate. Further improvements will likely come from a better description of the tails of the distribution, for which certain limit theorems ensure a universal behaviour, which may indeed be parametrised more accurately.

Alternatively, if one does not require a simple functional form, we have shown how to derive the exact affinity distribution using a characteristic function approach. In this context, further improvements will have to take into account higher order background models and positional dependencies. Here we have considered a zeroth order background model to derive the distribution *p*_ε_, and we assumed identically distributed affinities, *a_l_*, to simplify Eq. (8). Both approximations can, in principle, be replaced by a more complicated model, but the characteristic function approach would still apply.

On the numerical side, it would be worthwhile to consider a better implementation of the Fourier transformation (and its inverse) over unevenly discretized domains. Our simple FFT implementation is straightforward, but it cannot accurately account for the region of small affinities, where the cumulative distribution *Pr*[*x*>*A*]≈1. This is because small overall affinities derive from many terms, *a_i_*≈0, which are not well resolved in our discretisation. The conventional FFT algorithms use an equi-distant discretisation of both the distribution function and its Fourier transform. This puts a lower limit on the achievable granularity and therefore we generally loose information from sites with very small *a_i_*≈0. Fortunately, from a practical point of view, this region is also not very interesting, as one is mostly concerned about the regions with relatively high affinities.

Notice that we took the matrices provided by TRANSFAC at face value, and did not pre-process them in any way. Clearly, many matrices are rather unspecific with low information content and correspondingly high baselevel affinities. In a more refined analysis one would probably want to remove them prior to the analysis, for example by invoking a quality measure as in [11]. Moreover, there is a large redundancy which needs to be resolved at the end of the analysis. It would certainly be helpful to start with a non-redundant set of matrices, but any such derivation maybe more challenging than a simple post-processing.

In summary, the combination of the physical binding model and the statistical normalization brings our theoretical predictions a step closer to the real world. To our knowledge, this is the first attempt to provide a quantitative measure which ranks transcription factors for a given sequence, and which can be compared directly with large-scale binding data. Its successful application to human promoter sequences, serves as an encouraging example of how the method can be applied to other sequences.

## Supporting Information

Text S1 Supplementary material.
(0.00 MB TEX)

Figure S1 Number of promoters with rank r.
(1.24 MB EPS)
